# Effects of gestational low dose perfluorooctanoic acid on maternal and “anxiety-like” behavior in dams

**DOI:** 10.3389/ftox.2022.971970

**Published:** 2022-08-29

**Authors:** Alyssa K. Merrill, Katherine Conrad, Elena Marvin, Marissa Sobolewski

**Affiliations:** Department of Environmental Medicine, University of Rochester School of Medicine, Rochester, NY, United States

**Keywords:** perfluorooctanoic acid, maternal behavior, anxiety like behaviors, pregnancy, endocrine disrupting chemical (EDC)

## Abstract

Pregnancy is a unique critical window with nearly ubiquitous exposure to low concentrations of endocrine disrupting chemicals, such as per- and poly-fluoroalkyl substances (PFAS). Human and animal research suggests that PFAS compounds disrupt hypothalamic-pituitary-adrenal axis function, with some evidence of altered “anxiety-like” behavior, but little is known about the potential effects on maternal mental health following exposures during pregnancy. Evaluating the consequences of gestational PFAS exposures on maternal health is essential, because approximately 1 in 10 women experience postpartum depression, often with increased anxiety. To address this gap, dams were exposed to a low dose, 0.1 mg/kg, of perfluorooctanoic acid (PFOA) from gestational day 0 to birth. Maternal behavior was then observed from postnatal days 5–9, and “anxiety-like” behavior was measured using open field spontaneous locomotor behavior and elevated plus maze following weaning. No difference was observed in the litter size or sex of offspring. Gestational PFOA exposure altered maternal behavior. Despite similar nursing durations, PFOA dams spent more time nursing in a flat posture and on their side, and less time in kyphosis. Despite significantly quicker first contact, PFOA dams did not return pups to the nest quicker, indicating reduced retrieval latency. At weaning, dams displayed increased “anxiety-like” behaviors in the elevated plus maze with a significantly higher mean duration in the closed arms and reduced choice frequency with significantly lower number of entries in the closed and open arms. PFOA dams showed reductions in ambulatory movement across the session. Pregnancy exposure to PFOA altered both maternal and “anxiety-like” behavior in dams. Additional assays focused on depression-associated behaviors, such as forced swim, anhedonia, and social preference, will further delineate behavioral mechanisms. Further research on the effects of environmental contaminant exposures during pregnancy should investigate how co-exposures to other risk factors, such as stress, may enhance behavioral toxicity. Understanding how environmental contaminant exposure during pregnancy effects maternal depression-associated, and/or “anxiety-like” behavior is necessary for the public health protection of women.

## 1 Introduction

Pregnant women are ubiquitously exposed to per- and poly-fluoroalkyl substances (PFAS) (Stein et al., 2012; Braun et al., 2014; Kalloo et al., 2018; Louis et al., 2019; Eick et al., 2021), a chemical class containing thousands of compounds used in a multitude of consumer products and industrial applications, e.g. food packaging, waterproof clothing, and fire-fighting foams (Glüge et al., 2020). PFAS have been observed in rodent models to alter the hypothalamic-pituitary-adrenal axis and “anxiety-like” behaviors (Piekarski et al., 2020). One highly persistent PFAS in particular, perfluorooctanoic acid (PFOA) a synthetic stain and water repellent (Begley et al., 2005; Vierke et al., 2012), has been shown to increase “anxiety-like” behavior in adult male mice and gestationally exposed offspring (Johansson et al., 2008; Onishchenko et al., 2011; Shi et al., 2020; Wang Y. et al., 2021), to elevate corticotrophin-releasing factor in the brain of adult male mice (Wang Y. et al., 2021), and to raise serum corticosterone levels in adult female mice (DeWitt et al., 2009). Little research however has explored the potential effects of pregnancy PFOA exposure on pregnant rodents to help understand maternal susceptibility. One, recent study in pregnant women indicates that PFOA and perfluorononanoic acid modify stress responsivity by altering corticotropin-releasing hormone (Eick et al., 2021). A major gap exists in understanding the behavioral toxicity of PFOA in pregnant rodents, specifically with regard to stress related, “anxiety-like” behavioral domains.

Toxicological studies often utilize pregnancy exposures to understand the consequences of exposure on fetal health and development, ignoring the health of the mother. As a result, pregnancy is an understudied critical window for postpartum mental health. In rodents, physiological changes initiate maternal behavior (Rosenblatt et al., 1988; Catanese et al., 2015; Keller et al., 2019). Maternal behavior has profound consequences on the health and development of the offspring (Dulac et al., 2014). Exposure to environmental contaminants, such as bisphenols (Palanza et al., 2002; Kundakovic et al., 2013; Boudalia et al., 2014; Derouiche et al., 2015; Johnson et al., 2015; Catanese and Vandenberg, 2017; LaPlante et al., 2017), polychlorinated biphenyls (Cummings et al., 2005; Simmons et al., 2005), and herbicides (Stürtz et al., 2008), have been observed in rodent models to alter maternal behavior. PFOA in rodent studies has been shown to alter lactation and nursing (White et al., 2007; White et al., 2011). However, research is needed to investigate the broad maternal behavioral shifts and the potential consequences of PFOA on additional maternal behavioral deficits.

In addition to understanding effects on maternal behavior, research is needed to determine how these perturbations might impact the later life mental health of the mother. Women are two times more likely than men to experience mood disorders (Seney and Sibille, 2014). Incidence rates of anxiety, stress-related, and mood disorders in women are highest during pregnancy and postpartum leading to the hypothesis that pregnancy is a susceptible window for psychiatric disorders in women (Jacobson et al., 2021). Two mood disorders of concern, postpartum depression and postpartum anxiety are highly prevalent with rates as high as 11.5% in the United States (Ko et al., 2017) and 17.22% globally (Wang Z. et al., 2021), and 18% in the United States (Farr et al., 2014) and 33% globally (Grant et al., 2008), respectively. In pregnant women elevated corticotropin-releasing hormone levels have been associated with an increased risk of postpartum depression (Yim et al., 2009). A scoping epidemiological review has shown exposure to endocrine disrupting chemicals to be associated with female’s risk of mood disorders during pregnancy and postpartum (Jacobson et al., 2021), but research on PFOA is lacking.

To address these gaps, the current study assessed the effects of pregnancy exposure to PFOA on maternal and “anxiety-like” behavior in dams. Pregnant mice were exposed to a low-dose of PFOA from gestational day zero until birth. Following birth, maternal behavior was quantified, and pup retrieval, locomotor, and elevated plus maze behavioral assays were performed.

## 2 Materials and methods

### 2.1 Animals and husbandry

To determine whether perfluorooctanoic acid (PFOA) alters maternal behavior, adult (post-natal day (PND) 60) male and female C57BL/6J mice obtained from Jackson Laboratories (Bar Harbor, ME) were bred using a monogamous pairing scheme. The presence of a mucus plug was denoted as gestational day (GD) 0. If a mucus plug was observed, males were removed from the home cage. Pregnant dams (vehicle *n* = 13, PFOA *n* = 11) remained singly housed throughout weaning. All dams for either treatment group were counterbalanced to ensure any variation in the housing conditions was evenly distributed. Pregnant mice were exposed orally to either PFOA (0.1 mg/kg) or vehicle daily from GD 0 until birth. The dose was defined as a relatively low dose because it is at or below levels typically shown to cause effects in animal studies (Fenton et al., 2009; Agency, U.E.P., 2016). PFOA (96% pure, Sigma Aldrich, St. Louis, M.O.) was dissolved in Barnstead GenPure ultrapure water (Thermo Fisher Scientific, Waltham, M.A.) to produce a 0.125 mg/ml solution and 20 µl of the solution was injected into the abdomen of the mealworm. The PFOA solution concentration and volume injected were based off of the average gestational weight of 25 g. Following injection, the mealworm was flash frozen to prevent degradation and leakage. Dams were dosed via a PFOA-spiked mealworm at the same time every day from GD 0 until birth, with vehicle dams given a plain mealworm (Sobolewski et al., 2016). Cages were checked following mealworm administration to ensure the dams consumed the entire mealworm. Pregnancies were monitored daily to evaluate whether there were differences in litter size, or sex ratio of the offspring. On average, litters contained 6.71 ± 0.32 pups (Table 1). Dams were individually housed in a vivarium room maintained at 22 ± 2°C with a 12-h light-dark cycle (lights on at 06:00 h). Labdiet Rodent 5,010 diet and tap water were provided *ad libitum* throughout the entire experimental duration. For all behavioral testing, scorers were blinded to treatment group. Animals were counterbalanced by treatment group for behavioral run order to equalize potential temporal/run order effects. All behavioral testing occurred between hours of 1,200 and 1,800. For maternal behavior, care was taken to not disrupt the cage and scans were not conducted within the first few days of birth to minimize early disruptions. All experiments were carried out according to NIH Guidelines and the animal study was reviewed and approved by the University of Rochester Medical School University Committee on Animal Resources.

**TABLE 1:** Litter size and sex distribution.

Treatment	Litter size	*p*-value	Males	*p*-value	Females	*p*-value	Average sex ratio (F/M)	*p*-value	Stillborn pups [total (dams)]
PFOA	6.36 ± 0.46	0.33	3.55 ± 0.47	0.25	2.82 ± 0.47	0.85	0.72 ± 0.21	0.29	4 (3)
Vehicle	7.00 ± 0.43		4.30 ± 0.43		2.69 ± 0.44		0.80 ± 0.18		1 (1)

### 2.2 Maternal behavior

#### 2.2.1 Maternal behavior time scans

**FIGURE 1:**
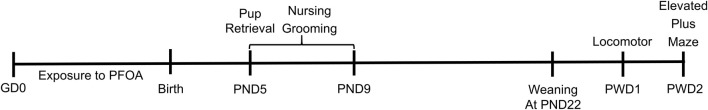
Experimental Timeline. Dams were orally exposed to PFOA or vehicle from gestational day (GD) 0 till birth. Following birth, maternal behavior was quantified over postnatal days (PNDs) 5–9, with pup retrieval assess on PND 5. After weaning on postweaning day (PWD) 1 dams underwent spontaneous open field locomotor activity assessment and on PWD 2 dams were assessed utiulizing the elevated plus maze assay.

Maternal behavior was observed from PND 5 to 9 (Figure 1). Dams with their pups were observed in their home cages in the vivarium between the hours of 1,400–1,700. Point scans were conducted for 1 minute, every 10 minutes, over the course of 1 h. If a behavior occurred during the observation a score of one was given to that behavior. If a behavior did not occur a score of zero was given. Multiple behaviors could occur within the behavioral scan. Behaviors were scored using a modified ethogram (Palanza et al., 2002; Stern et al., 2002). Definitions included: Nursing - pup(s) attached to the nipples of the dam. Nursing was further classified based off of dam posture (Stern et al., 2002). Nursing was sub-categorized in the following ways: Flat - dam laying over the pup(s), which are attached to nipples, with the back and head in one plane. Side - dam is laying on a side of the torso, with pup(s) attached to the nipples, the dam is not covering the pup(s) with her body. Sitting - dam is on hind legs with the pup(s) attached to the lower nipples, pup(s) are not covered by the dam. Kyphosis - dam has an upright dorsal arch posture over the pup(s), which are attached to the nipples. Adjusting - dam is either adjusting pup(s) on the nipples and/or moving in a circular motion within the nest with pup(s) remaining attached to her nipples. In addition to nursing, maternal behaviors scored included: Grooming - either the dam licking and/or grooming herself or the pup(s). Eating—dam was actively eating food pellets from the hopper and/or drinking water from the glass water bottle. Location—whether the dam was inside or outside of the nest was denoted.

#### 2.2.2 Pup retrieval

Pup retrieval was conducted on PND 5. Dams and pups were habituated in the home cage to the testing environment for 30 minutes prior to running the assay. Upon testing the dam was removed from the home cage and three pups were randomly selected from the nest. If possible, based off of the sex distribution of the litter, at least one male and one female pup were used. The three selected pups were placed in the three corners of the home cage not occupied by the nest. If pup(s) remained they were placed back in the home cage nest. Then, the dam was placed into the center of the home cage and given 10 minutes to retrieve and return all pups to the nest. If the dam did not retrieve all pups at the end of the 10 minutes the pups were returned to the nest by the experimenter. Latency to first contact (e.g. sniff or attempt to pick-up the pup), latency to retrieve the first pup into the nest, and latency from first contact to retrieval into the nest were scored by an observer blind to treatment status utilizing Observer XT 13 (Noldus, Leesburg, V.A.).

### 2.3 Elevated plus maze

Two days after weaning dams underwent elevated plus maze. The elevated plus maze apparatus had two open arms (no sides; 34.9 × 6.07 cm), two closed arms (with sides; 34.9 × 6.07 × 19.13 cm), and a central platform (6.1 × 6.1 cm). The apparatus, made of black plastic, was placed 93.73 cm above the floor. The dam was placed in the center of the apparatus to start and allowed to explore for 5 minutes. Sessions were video recorded and the amount of time spent in, the entries, and the mean average duration in the open arms, closed arms, and center was scored by an observer blind to treatment status utilizing Observer XT 13 (Noldus, Leesburg, V.A.).

### 2.4 Locomotor

One day following weaning dams underwent spontaneous open field locomotor activity assessment. Locomotor activity was measured in chambers (27.3 × 27.3 × 20.3 cm) equipped with 48-channel infrared photobeams (Med Associates Inc., St. Albans, V.T.). Photobeam breaks were recorded for 1 h and aggregated into 5 min intervals to assess stereotypic, vertical, and ambulatory counts, as well as ambulatory distance and time in zone. Ambulatory counts were defined as the number of photobeam breaks while in an ambulatory movement. Ambulatory distance was defined as the differences in angular movements. Stereotypic counts were defined as the quantity of beam breaks in a 2 × 2 in photobeam array box that were not ambulatory counts, therefore movements that occurred while the animal remained within a localized space. Vertical counts were defined as the total time spent breaking any photobeams in the *z*-axis, which was defined by photobeams 7 cm above the floor of the locomotor box. Time in zone was defined as the total time spent within a given zone. Zone entries were defined as the total entries into a given zone. All parameters were broken down by the center versus the edge of the chamber. The edge was defined as the space in between the outer (25.6 × 25.6 cm) and inner (15.7 × 15.7 cm) square of the chamber. The center was defined as all remaining space not in the edge, which was a 15.7 × 15.7 cm square in the center of the chamber.

### 2.5 Statistical analysis

Maternal behaviors, pup retrieval, elevated plus maze, and whole session locomotor performance were analyzed with a student’s t-test. For unidirectional hypotheses, one-sided student’s t-test were used. Dams who failed to retrieve a single pup into the nest over the course of observation were excluded from analysis as these dams failed to complete the assay (Vehicle *n* = 2, PFOA *n* = 1). Repeated measures ANOVAs were used to assess the spontaneous open field locomotor activity across the 5-min intervals for the 1-h session with individual subject identifiers set as a random effect with time (5 min intervals), treatment, and the time by treatment interaction assessed. Locomotor data were further analyzed separating by zone: center and edge activity. Behavioral correlations were assessed using a Pearson’s correlation. Outliers were removed following a statistically significant Grubb’s test (Graphpad Software Inc., San Diego, C.A.). One dam out of 24 was removed due to freezing for more than 30% of the time on the elevated plus maze (Grant et al., 2008), three data points out of 288 data points were removed from the maternal behavior time scans, and 78 data points out of 4,992 data points were removed from the spontaneous open field locomotor assay. No more than one data point was ever removed by treatment by endpoint. Statistical analyses were conducted using JMP Pro 16.0 (SAS Institute Inc., Cary, N.C., United States). *p* values ≤0.05 were considered statistically significant, while marginally non-significant values (*p* values ≤0.10) are also indicated.

## 3 Results

### 3.1 Maternal behavior time scans

#### 3.1.1 Nursing

**FIGURE 2:**
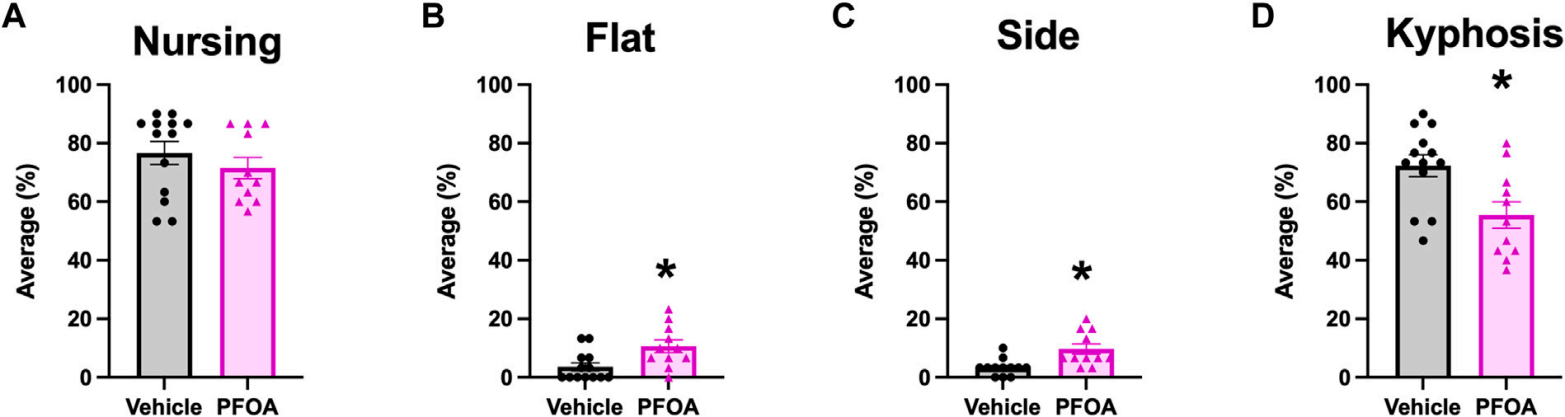
Dams Nursing Activity. Average amount of time over postnatal days 5–9 that dams spent nursing **(A)**, nursing in a flat posture **(B)**, nursing in a side posture **(C)**, and nursing in a kyphosis posture **(D)**. Data represented as mean ± standard error of the mean, *n* = 11/PFOA and n = 12-13/vehicle. Data analyzed with two-sided student’s t-test. Asterisks means *p* ≤ 0.05.

**FIGURE 3:**
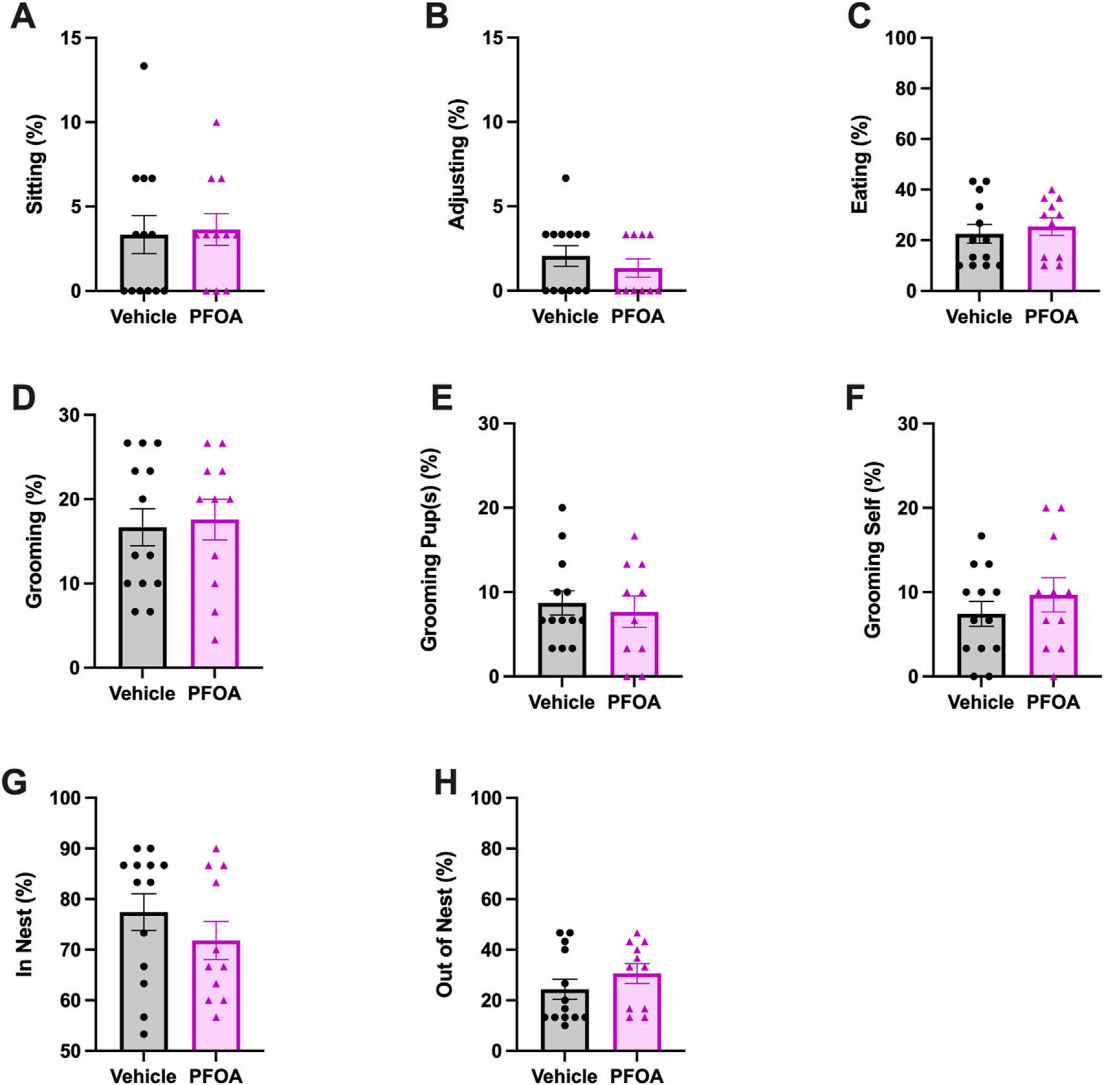
Maternal Behavior Averages. The average activity of each maternal behavior sitting **(A)**, adjusting **(B)**, eating **(C)**, grooming **(D)**, grooming pup(s) **(E)**, grooming self **(F)**, in nest **(G)**, and out of nest **(H)** scored over the course of postnatal days 5–9. Data represented as mean ± standard error of the mean, *n* = 10-11/PFOA and n = 13/vehicle. Data analyzed with two-sided student’s t-test.

Although PFOA dams did not differ in the average amount of time spent nursing (*t* = 0.95, *p* = 0.35; Figure 2A) the posture of nursing differed. Overall, PFOA dams spent significantly more time on average nursing in flat and side postures, with PFOA dams spending 7.02% more time in a flat posture (*t* = −2.83, *p* = 0.0098; Figure 2B) and 6.36% more time in a side posture (*t* = −3.36, *p* = 0.0030; Figure 2C) than vehicle dams. Furthermore, PFOA dams on average spent 16.85% less time in kyphosis compared to vehicle dams (*t* = 2.90, *p* = 0.0084; Figure 2D). No difference was present in the average duration PFOA dams spent in a sitting posture (*t* = -0.20, *p* = 0.84; Figure 3A) or adjusting (*t* = 0.86, *p* = 0.40; Figure 3B) while nursing.

#### 3.1.2 Eating

PFOA dams did not differ in the time spent eating compared to vehicle dams on average (*t* = −0.56, *p* = 0.58; Figure 3C).

#### 3.1.3 Grooming

There was no difference in the amount of time PFOA dams spent on overall grooming on average (*t* = −0.27, *p* = 0.78; Figure 3D). Nor was a difference present in the duration of time PFOA dams spent grooming pup(s) (*t* = 0.45, *p* = 0.65; Figure 3E), or themselves (*t* = −0.92, *p* = 0.37; Figure 3F) on average.

#### 3.1.4 Location within home cage

There was no difference in the average amount of time that PFOA dams spent in the nest (*t* = 1.07, *p* = 0.30; Figure 3G) or out of the nest (*t* = −1.10, *p* = 0.28; Figure 3H).

### 3.2 Pup retrieval

**FIGURE 4:**
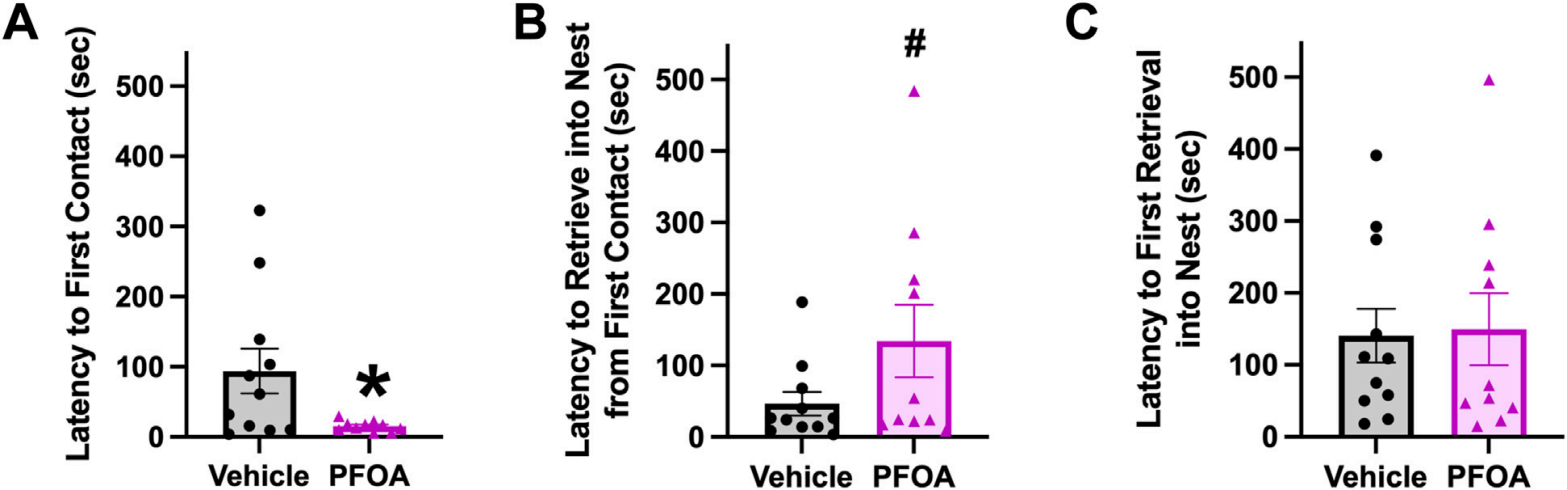
Pup Retrieval. Pup retrieval was performed on postnatal day 5. Latency to first contact **(A)**, latency to retrieve into nest from first contact **(B)**, and latency to first retrieval into nest **(C)** were measured. Data shown as mean ± standard error of the mean, *n* = 10/PFOA and *n* = 11/vehicle. Data analyzed with two-sided student’s t-test. Asterisks means *p* ≤ 0.05 and number sign means *p* ≤ 0.10.

PFOA dams made the first contact with a pup 78.52 s quicker than vehicle dams (*t* = 2.34, *p* = 0.030; Figure 4A). Although no difference in time to retrieve the first pup to the nest was present (*t* = −0.15, *p* = 0.88; Figure 4C), PFOA dams spent 87.58 s longer from first contact with a pup to retrieve the first pup to the nest than vehicle dams (*t* = −1.71, *p* = 0.10; Figure 4B).

### 3.3 Elevated plus maze

**FIGURE 5:**
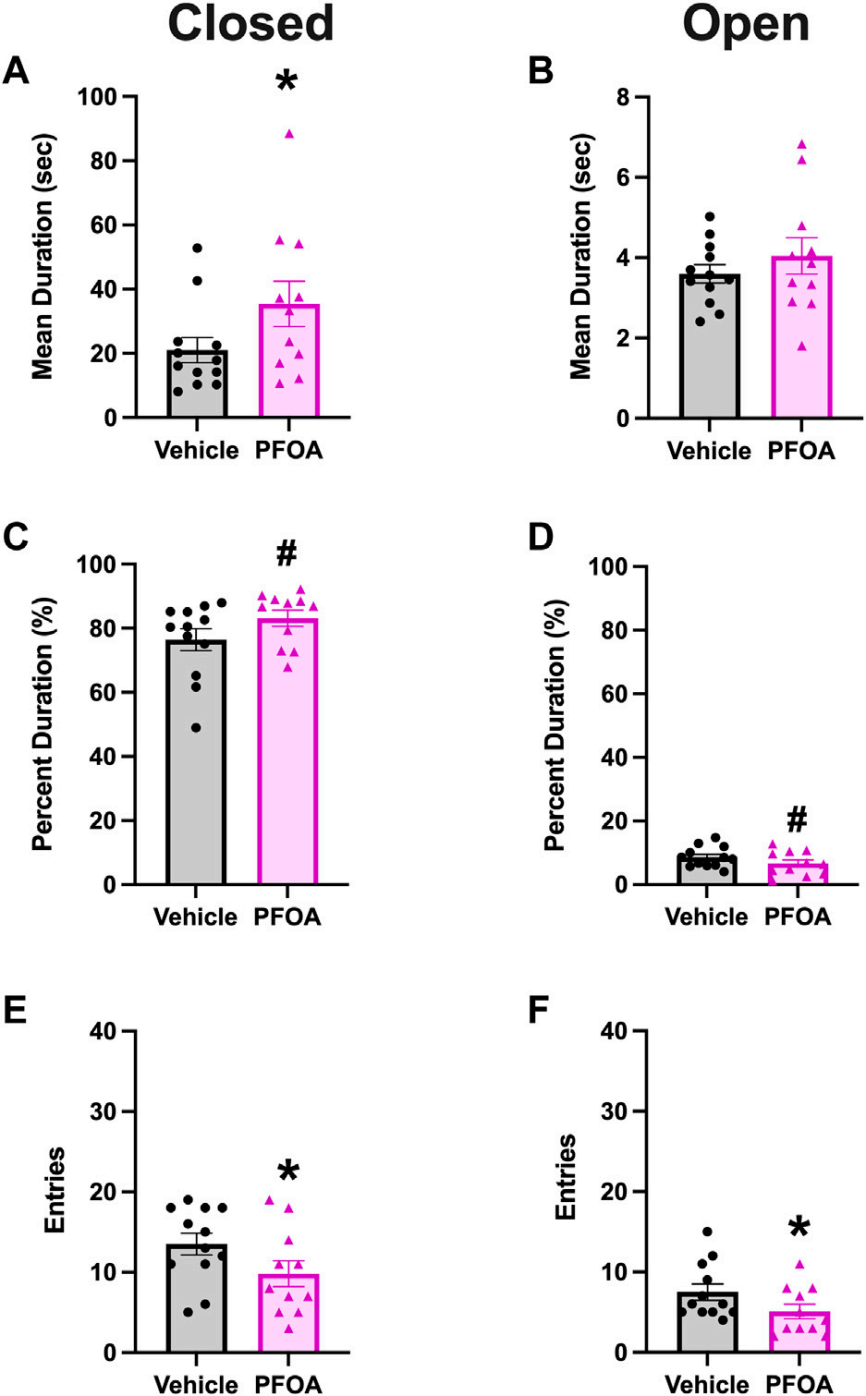
Elevated Plus Maze. PFOA dams had a significantly higher mean duration **(A)** and a marginally non-significantly elevated percent duration **(C)** in the closed arms. Additionally, PFOA dams had a marginally non-significantly decrease in the percent duration **(D)** spent in the open arms. Furthermore, the number of entries into both the closed arms **(E)** and open arms **(F)** were significantly reduced in PFOA dams. There was no difference in the mean duration **(B)** spent in the open arms. Data shown as mean ± standard error of the mean, *n* = 11/PFOA and *n* = 12/vehicle. Data analyzed with one-sided student’s t-test. Asterisks means *p* ≤ 0.05 and number sign means *p* ≤ 0.10.

PFOA dams had a significantly higher mean duration in the closed arms (*t* = −1.83, *p* = 0.041; Figure 5A). Furthermore, PFOA dams spent 83.16% of time in the closed arms, compared to only 76.46% in the vehicle dams (*t* = −1.54, *p* = 0.070; Figure 5C). Correspondingly, PFOA dams had a marginally non-significant decrease in the percent duration spent in the open arms (*t* = 1.31, *p* = 0.10; Figure 5D). PFOA dams also had a significantly lower number of entries into both the closed (*t* = 1.76, *p* = 0.046; Figure 5E) and open (*t* = 1.77, *p* = 0.045; Figure 5F) arms. There was no difference in the mean duration (*t* = -0.90, *p* = 0.19; Figure 5B) that PFOA dams spent in the open arms.

### 3.4 Locomotor

**FIGURE 6:**
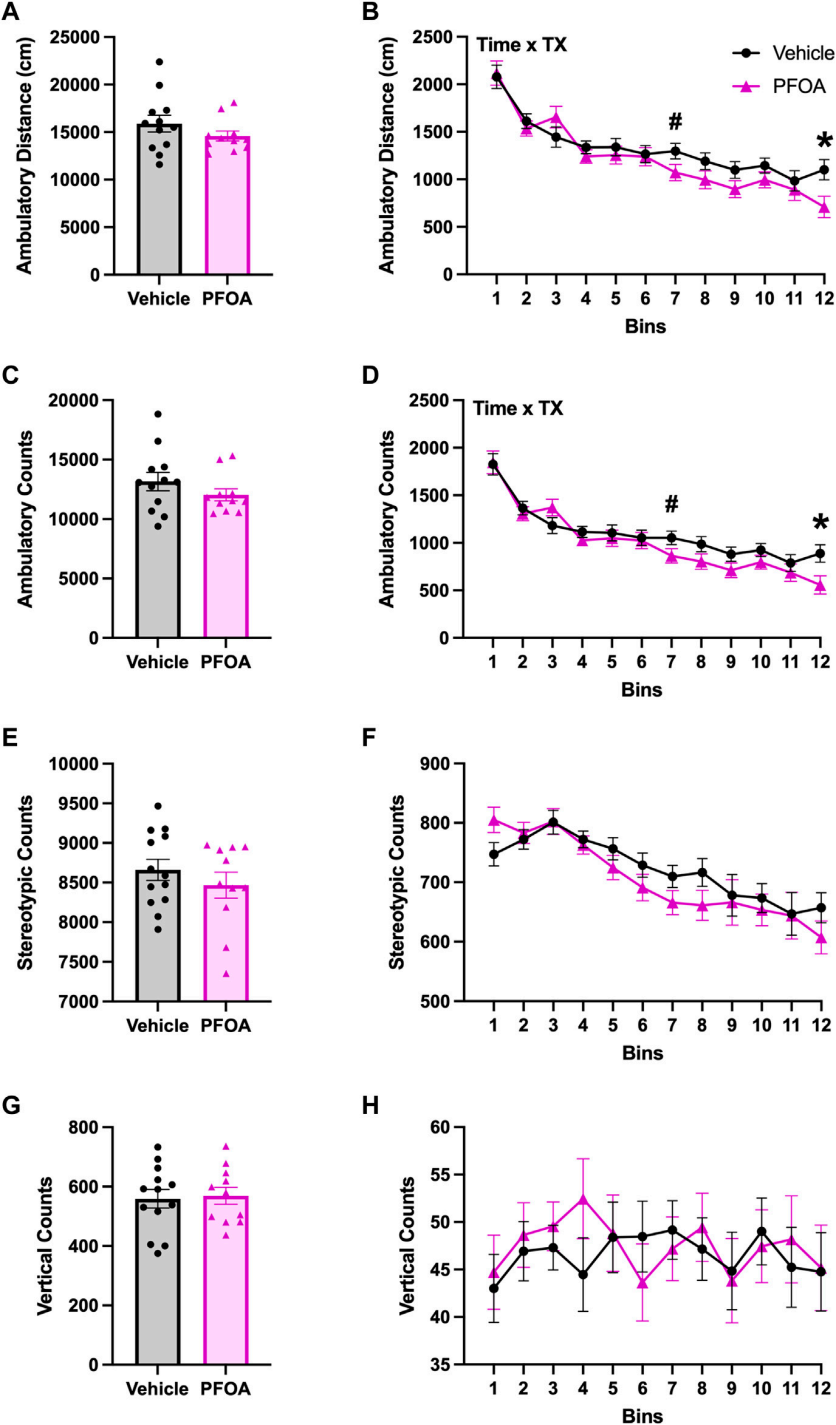
Spontaneous Open Field Locomotor. One day after weaning of the pups spontaneous open-field locomotor activity was assessed in the dams. PFOA dams had no overall whole session differences in ambulatory distance **(A)**, ambulatory counts **(C)**, stereotypic counts **(E)**, or vertical counts **(G)**. Ambulatory distance **(B)** and ambulatory counts **(D)** had significant time by treatment interactions present with reductions in ambulation. No time by treatment interactions were observed in stereotypic counts **(F)**, or vertical counts **(H)**. Data shown as mean ± standard error of the mean, *n* = 11/PFOA and *n* = 12-13/vehicle. Data analyzed with either two-sided student’s t-test or repeated measures ANOVAs followed by two-sided student’s t-test. Asterisks means *p* ≤ 0.05 and number sign means *p* ≤ 0.10.

**FIGURE 7:**
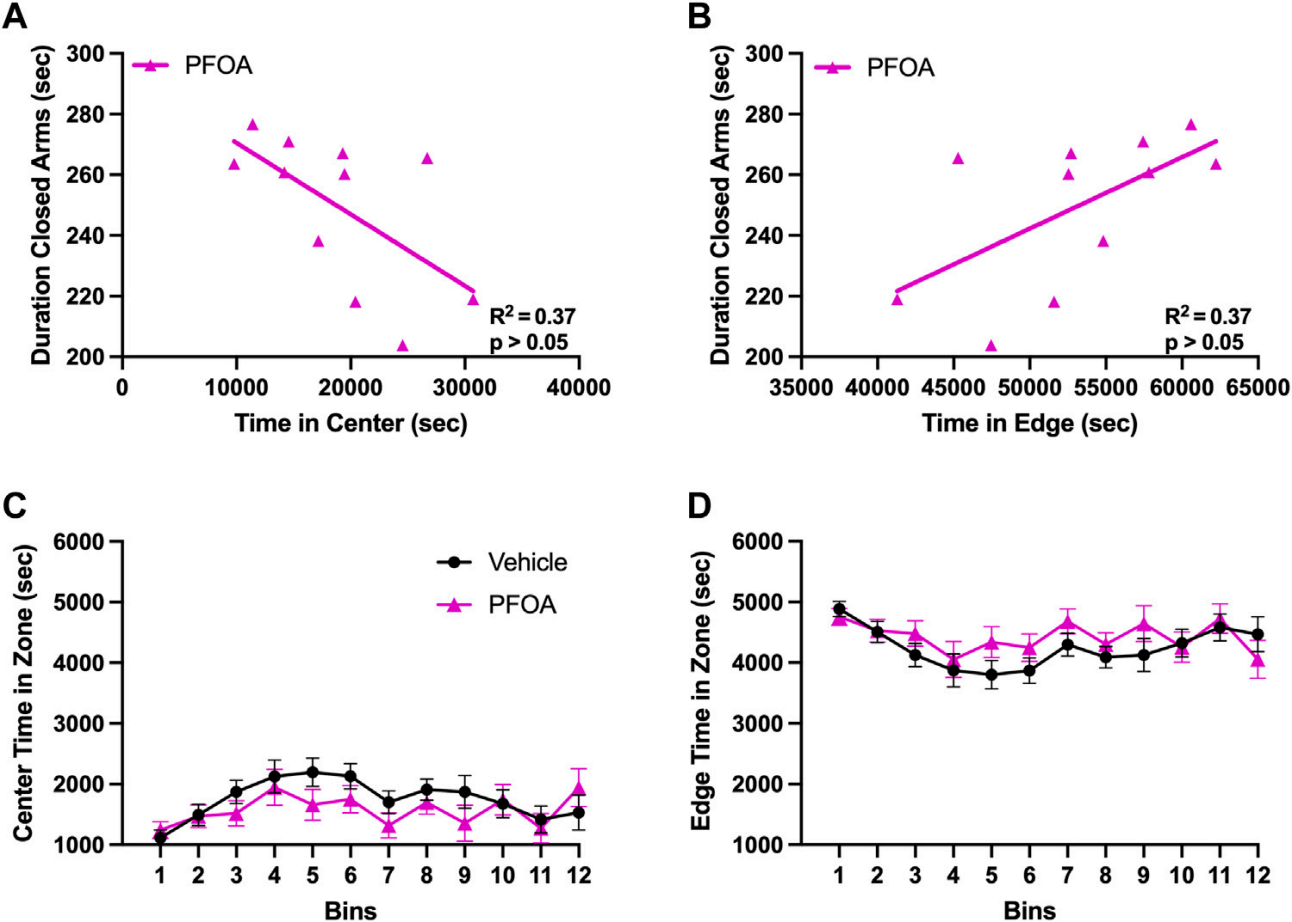
Closed Arm Duration in Relation to Locomotor Location. The total duration of time PFOA dams spent in the closed arms of the elevated plus maze was negative correlated with the duration of time spent in the center of the locomotor chamber **(A)**. Furthermore, the amount of time PFOA dams spent in the closed arms of the elevated plus maze was positively correlated with the duration of time spent in the edges of the locomotor assay **(B)**. No difference was observed in the time PFOA dams spent in either the center **(C)** or the edge **(D)** of the locomotor chamber. Graphs C and D data shown as mean ± standard error of the mean, *n* = 11/PFOA and n = 13/vehicle. Data analyzed with either a Pearson correlation coefficient or repeated measures ANOVAs.

PFOA dams had significant time by treatment interactions in the whole session ambulatory distance (F (11,231) = 1.89, *p* = 0.042) with significantly reduced ambulatory distance at bin 12 (*t* = 2.51, *p* = 0.020) and marginally non-significant reductions in ambulatory distance at bin 7 (*t* = 1.91, *p* = 0.070; Figure 6B). Additionally, PFOA dams had a significant time by treatment interaction in ambulatory counts (F (11,231) = 1.86, *p* = 0.046) with significantly reduced ambulatory counts at bin 12 (*t* = 2.47, *p* = 0.022) and marginally non-significant reductions in ambulatory counts at bin 7 (*t* = 1.81, *p* = 0.085; Figure 6D) compared to vehicle dams. PFOA dams did not differ from vehicle dams in whole session time by treatment interactions in stereotypic counts (F (11,242) = 0.99, *p* = 0.46; Figure 6F) or vertical counts (F (11,242) = 0.60, *p* = 0.82; Figure 6H). PFOA dams also did not differ from vehicle dams in time by treatment interactions in the center ambulatory distance (F (11,231) = 1.19, *p* = 0.30; Supplementary Figure S1B), ambulatory counts (F (11,231) = 1.28, *p* = 0.24; Supplementary Figure S1F), stereotypic counts (F (11,242) = 1.29, *p* = 0.23; Supplementary Figure S1J), vertical counts (F (11,242) = 0.68, *p* = 0.76; Supplementary Figure S1N), time in zone (F (11,242) = 1.23, *p* = 0.27; Figure 7C), or zone entries (F (11,242) = 1.26, *p* = 0.25; Supplementary Figure S1R). Furthermore, PFOA dams did not differ in time by treatment interactions compared to vehicle dams in the edge ambulatory distance (F (11,231) = 1.62, *p* = 0.093; Supplementary Figure S1D), ambulatory counts (F (11,231) = 1.60, *p* = 0.10; Supplementary Figure S1H), stereotypic counts (F (11,242) = 0.90, *p* = 0.55; Supplementary Figure S1L), vertical counts (F (11,242) = 0.92, *p* = 0.53; Supplementary Figure S1P), time in zone (F (11,242) = 1.23, *p* = 0.27; Figure 7D), or zone entries (F (11,242) = 1.77, *p* = 0.059; Supplementary Figure S1T).

PFOA dams did not have any whole session locomotor differences compared to vehicle dams in ambulatory distance (*t* = 1.23, *p* = 0.24; Figure 6A), ambulatory counts (*t* = 1.18, *p* = 0.25; Figure 6C), stereotypic counts (*t* = 0.92, *p* = 0.37; Figure 6E), or vertical counts (*t* = −0.24, *p* = 0.81; Figure 6G). Additionally, PFOA dams did not differ from vehicle dams in the whole session center ambulatory distance (*t* = 1.53, *p* = 0.14; Supplementary Figure S1A), ambulatory counts (*t* = 1.46, *p* = 0.16; Supplementary Figure S1E), stereotypic counts (*t* = 0.90, *p* = 0.38; Supplementary Figure S1I), vertical counts (*t* = 0.62, *p* = 0.54; Supplementary Figure S1M), time in zone (*t* = 0.82, *p* = 0.42; data not shown), or zone entries (*t* = 1.45, *p* = 0.26; Supplementary Figure S1Q). Furthermore, PFOA dams did not differ from vehicle dams in the whole session edge ambulatory distance (*t* = 0.67, *p* = 0.51; Supplementary Figure S1C), ambulatory counts (*t* = 0.74, *p* = 0.47; Supplementary Figure S1G), stereotypic counts (*t* = -0.35, *p* = 0.73; Supplementary Figure S1K), vertical counts (*t* = -0.72, *p* = 0.48; Supplementary Figure S1O), time in zone (*t* = -0.82, *p* = 0.42; data not shown), or zone entries (*t* = 1.13, *p* = 0.27; Supplementary Figure S1S).

Although there was no difference in the center and edge zones of the locomotor behavior, the duration PFOA dams spent in the closed arms of the elevated plus maze was negatively correlated with the amount of time spent in the center of the locomotor chamber (*p* = 0.048, *R* ^2^ = 0.37; Figure 7A) and positively correlated with the duration of time spent in the edge of the locomotor chamber (*p* = 0.048, *R* ^2^ = 0.37; Figure 7B). No correlation was observed between the duration spent in the closed arms of the elevated plus maze with either time spent in the center (*p* = 0.60, *R* ^2^ = 0.029; data not shown) or in the edge (*p* = 0.60, *R* ^2^ = 0.029; data not shown) of the locomotor chamber in vehicle dams.

## 4 Discussion

Pregnancy is a period of significant physiological change required to maintain and support fetal development, initiate lactation, and prepare for parental care (Rosenblatt et al., 1988; Catanese et al., 2015; Keller et al., 2019). These changes are coordinated by changes in circulating hormones (estrogens and glucocorticoids) known to be targeted by endocrine disrupting chemicals, including per- and poly-fluoroalkyl substances. Relatively few rodent and epidemiological studies have investigated and/or shown that maternal behavior and postpartum depression risk are altered by pregnancy exposure to endocrine disrupting chemicals (Catanese et al., 2015; Jacobson et al., 2021). This is a relatively understudied critical window. More research is needed, as rodent models have not assessed the potential consequences of maternal exposure to a highly persistent contaminant, PFOA, on broad maternal and “anxiety-like” behavior. The present study investigated the effects of low-dose pregnancy PFOA exposure on maternal and elevated plus maze behavior. PFOA dams displayed altered nursing posture, increased passive nursing and decreased active nursing, as well as decreased latency to first contact. Furthermore, dams had a higher mean duration in the closed arms of the elevated plus maze and a decrease in entries into both the closed and open arms, indicative of increased “anxiety-like” behavior.

The present study found PFOA dams displayed reduced maternal behavior, indicated by an increase in passive nursing postures (Catanese et al., 2015). Though there was no difference in total time spent nursing the quality of nursing posture was altered by PFOA exposure. On average PFOA dams spent less time in kyphosis, an active nursing posture associated with greater milk production (Lonstein et al., 1998), and more time in passive flat and side nursing postures. Developmental studies focused on PFOA toxicity of the developing fetus should consider effects on nursing behavior and potentially, milk production (White et al., 2007; White et al., 2011). PFOA dams also had a reduction in latency to first contact with the pup and a marginal increase in latency from first contact to retrieval. Interestingly despite a significantly decreased latency to first contact, PFOA dams did not return their pups to the nest faster, indicative of delayed identification of the pups or a delay in the choice to return to the nest. Although nursing posture and pup retrieval were altered by PFOA exposure, no difference was observed in grooming. Licking and grooming of the pups shortly after birth is essential to remove the amniotic fluid and membranes to stimulate breathing in the pups and to prevent the pups from sticking to one another, to initiate urination and defecation, and to stimulate pup growth (Kuroda et al., 2011; Touhara, 2013). By targeting our maternal behavioral observations between postnatal days 5–9, potential effects of gestational PFOA exposure on early maternal licking and grooming behavior may have been missed. Previous research indicates that maternal exposure to PFOA may alter lactation, future research should investigate how alterations in nursing behavior interact with altered lactation and fetal development (White et al., 2007; White et al., 2011). As the differences in nursing behavior might be a consequence of altered lactation.

In addition to altered maternal nursing posture and pup retrieval, PFOA dams displayed changes in elevated plus maze, indicating increased preference for closed arms. PFOA dams spent a greater mean duration in the closed arms, spent marginally more time in the closed arms and less time in the open arms, and had reduced closed and open arm entries, behaviors indicative of increased “anxiety-like” behavior. Furthermore, in the spontaneous open field locomotor PFOA dams had significant time by treatment interactions for ambulatory distance and counts, with PFOA dams showing significant decreases in activity in the final activity bin. The ambulatory hypoactivity and decreased time to habituation are behaviors suggestive of disruptions in exploration. To separate these behavioral mechanisms, future research should evaluate locomotor behavior over multiple days or investigate home-cage locomotor behavior. Additionally, future behavioral tests should be conducted to expand our behavioral assessment of depression-associated and anxiety-like behavioral profiles, including but not limited to forced swim (depression), splash test (depression), repeated spontaneous locomotor assessments (anxiety), social novelty (anxiety), and sucrose preference (anhedonia), all of which share endocrine and neurochemical targets (Insel, 2014; Woody and Gibb, 2015). Increased anxiety is a symptom that may be observed in those with mood disorders, such as postpartum depression (Association, A. P., 2013). These alterations in elevated plus maze and locomotor activity indicate subtle alterations in fear mediated behaviors. Future studies need to evaluate these effects in the presence and absence of non-chemical stressors to determine how cumulative risk factors may enhance behavioral toxicity. Prior epidemiological research indicated that second trimester PFOA levels were not associated with postpartum depression risk up to 8 years postpartum (Vuong et al., 2020). However, in other epidemiological studies gestational PFAS levels are associated with altered stress physiology, elevated corticotropin-releasing hormone, dependent on self-reported stress (Eick et al., 2021) and cortisol levels (Goudarzi et al., 2017; Dreyer et al., 2020), in mothers. To help clarify the difference in epidemiological findings, future rodent and epidemiological research are needed to assess the effects of PFOA exposure and maternal stress during pregnancy to determine the effects of PFOA on depression and anxiety-related behaviors in a bi-directional and translational fashion.

The limitations of the present study include the time at which maternal behavioral observations began. Initial maternal licking and grooming, nursing, pup grouping, and time spent on the nest are essential for pup survival. By starting our maternal behavior observations on postnatal day 5 the current study may have missed possible differences in the initiation of maternal behavior. Additionally, when assessing pup retrieval ultrasonic vocalizations were not measured. Although both sexes were utilized to account for the sex differences in ultrasonic vocalizations PFOA exposure might have altered the vocalizations in the pups, delaying the dams’ recognition of the pups (D’Amato et al., 2005; Mogi et al., 2017). To determine whether delays in pup retrieval are due to alterations in maternal behavior or altered behavioral responses to ultrasonic vocalizations, ultrasonic vocalizations should be assessed during pup retrieval. Furthermore, only maternal and “anxiety-like” behaviors were evaluated. To gain a deeper understanding into the alterations of PFOA on the maternal nervous system biochemical outcomes, e.g. serum corticosterone levels and neurotransmitter concentrations in the brains of dams should be investigated. Additionally, future studies should explore the consequences of PFOA on pregnancy, e.g. fetal resorptions, attrition, parturition, and others. Also, the effects of PFOA on maternal and “anxiety-like” behavior were only assessed in C57BL/6J mice. The consequences of pregnancy PFOA exposure need to be investigated in other strains and species to identify strain and species specific effects. Additionally, no concurrent non-pregnant females were exposed. Future research should directly assess the influence of pregnancy alone on these behaviors. Moreover, the kinetics of the treat-based oral administration were not measured. As the method of oral administration, gavage, treat based administration methods (spiked mealworm, pellets, cookies, etc.), and/or other oral administration methods, might differ the kinetics future research should determine if different oral administration methods have similar PFOA kinetics. Furthermore, PFOA was not measured in either the dosing solution or the serum of the dams. Future research should analyze the serum concentration of PFOA across the dams’ lifespan to help with translation to humans. Lastly, this experiment tests the “anxiety-like” behaviors in dams immediately following weaning of the pups. Additional timepoints after birth and protracted timepoints are needed. The potential effects of exposure on later life maternal “anxiety-like” behaviors remains unknown. As mentioned, research in humans suggests that the effects of PFAS on stress regulation is modulated by social and economic stressors (Richardson et al., 2014). Again, future research is needed to determine the extent these effects can be enhanced by combinatorial exposures to stress.

Although pregnancy PFOA exposure altered maternal and “anxiety-like” behavior in dams, when translating animal research to human behavior, it is important to minimize reductionist interpretations that blame maternal behavior for alterations in fetal development. Human development is influenced by complex interactions of protective and detrimental risk factors (Richardson et al., 2014). Unlike most rodents, in humans mothers are not the sole contributor to offspring care, rather childcare is biparental, and supported by extended family networks with community support. The maternal and paternal contributions, broad social support, and societal resources need to be considered when investigating the effects of parenting on offspring health and development. The development of maternal behavior is complex, while gestational exposures to environmental contaminants can alter maternal behavior and postpartum depression risk (Catanese et al., 2015; Jacobson et al., 2021), so can a plethora of other intertwined genetic, lifestyle, socioeconomic, and environmental factors that are poorly understood (Boutté et al., 2021; Dubey et al., 2021; Cho et al., 2022; Docherty et al., 2022; Gastaldon et al., 2022). Research is needed to understand how these factors modulate associations between gestational environmental contaminant exposures and the risk for mood disorders following pregnancy. Furthermore, more research is needed to understand how EDC exposures during pregnancy change the brain and behavior of pregnant women, and all people who can become pregnant. This includes building better animal models and improving our translational relevance to human health.

In conclusion, pregnancy exposure to a highly prevalent and persistent PFAS, PFOA, modified maternal nursing posture, increased latency to first pup contact, and increased “anxiety-like” behavior. These data support the possibility that exposure to environmental contaminants may influence maternal and “anxiety-like” behavior. More research is needed to understand the effects of environmental contaminant exposures during pregnancy on the maternal nervous system and the persistency of these effects.

## Data Availability

The raw data supporting the conclusion of this article will be made available by the authors, without undue reservation.
