# Supplementary figures and images for: Effects of gestational low dose perfluorooctanoic acid on maternal and “anxiety-like” behavior in dams
Source: Front Toxicol. 2022 Aug 29;4:971970. doi: 10.3389/ftox.2022.971970 (PMC9464925; doi:10.3389/ftox.2022.971970)

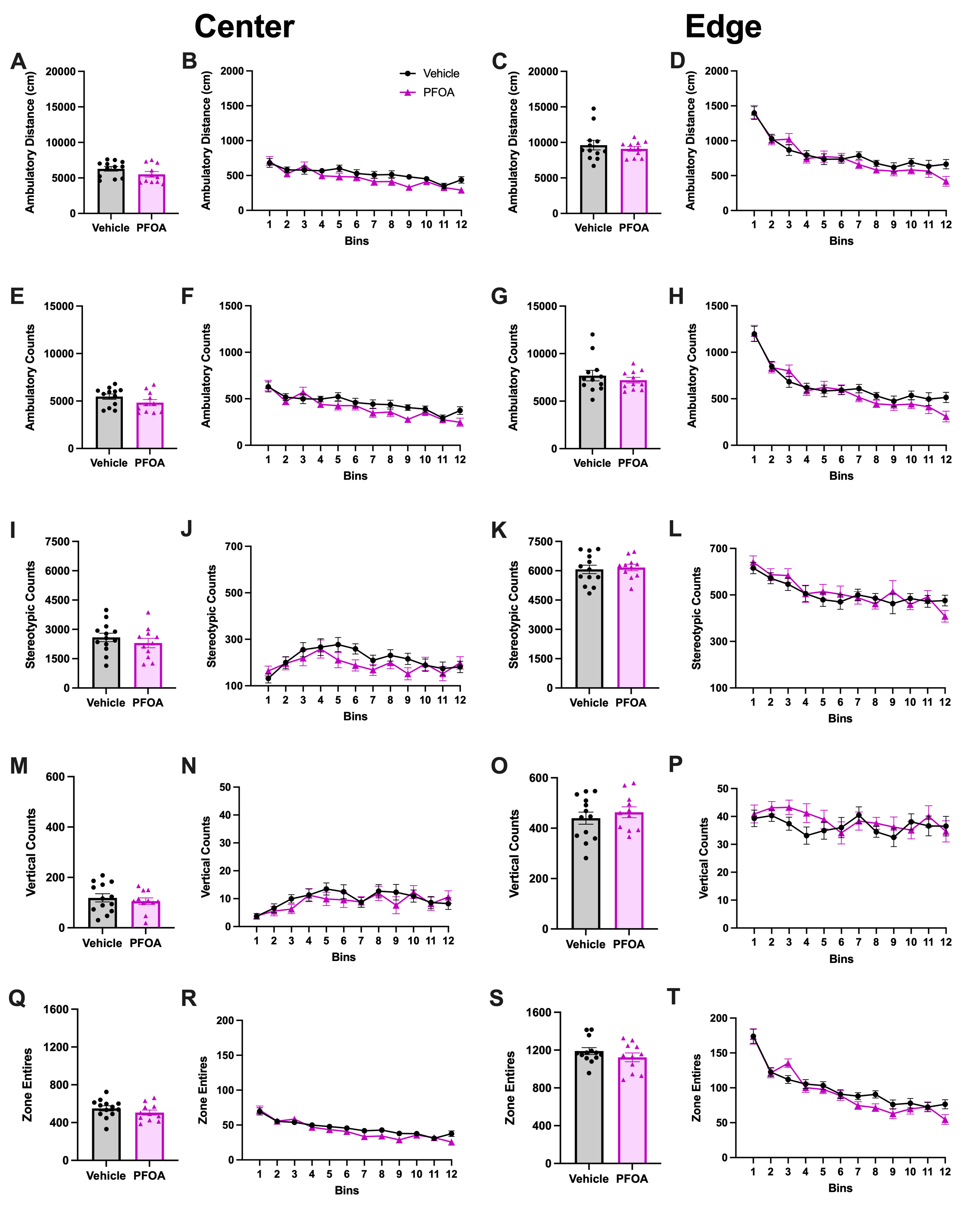

Supplement: Supplementary file 1 [file Image1.tiff]
